# The Influence of Speech Disturbances on Psychical Development

**Published:** 1899-09

**Authors:** 


### The Influence of Speech Disturbances on Psychical Development.

Alb. Liebmann (*Deutsche medicinal. Ztg.*, 1898, *xix.*, 157) claims that the view that disturbances of speech in children are only of little importance is altogether wrong. Should such a disturbance be of long duration and extend over the developmental stage (fourth or fifth year of life), grave dangers will threaten psychical development, and Liebmann enumerates many cases from his own practice bearing on this statement. In the first place the delicate mind of the patient, which in many of these cases is not altogether normal, is often greatly depressed by brooding over their speech defect; from the scolding and whipping often given them by their teachers, and by taunts of their comrades. The degree to which the mind is affected is by no means always in proportion to the degree of speech defect, and we meet quite frequently with marked psychic depression in cases where the speech defect is quite unimportant, perhaps only amounting to an esthetic defect. On the other hand, the psychic symptoms are always dependent on the attitude of the parents, teachers and play-fellows; recrimination, scolding, taunting, are sufficient to distress the childish mind in a most violent manner in cases where only a very slight speech disturbance is present. Stuttering and stammering persons are especially threatened, and Liebmann met with severe psychic symptoms accompanying even where the person was affected with only very little stammering. Through the removal of the speech defect the former may frequently be abolished at once, while those cases in which this affection had been neglected in childhood, the patient frequently suffers for decades from colossal psychical depression, which may even lead to suicide. This therapeutic interference at the earliest possible stage is also absolutely necessary, for the reason that the defect of speech is also of the greatest consequence in the education of the intellectual faculties, and to postpone the treatment will be bitterly regretted, as the retardation of the mental development progresses and treatment becomes more and more difficult.

